# Genetic dissection of Rift Valley fever pathogenesis: *Rvfs2* locus on mouse chromosome 11 enables survival to early-onset hepatitis

**DOI:** 10.1038/s41598-020-65683-w

**Published:** 2020-05-26

**Authors:** Leandro Batista, Gregory Jouvion, Dominique Simon-Chazottes, Denis Houzelstein, Odile Burlen-Defranoux, Magali Boissière, Satoko Tokuda, Tania Zaverucha do Valle, Ana Cumano, Marie Flamand, Xavier Montagutelli, Jean-Jacques Panthier

**Affiliations:** 1 Mouse Functional Genetics, Institut Pasteur, UMR3738, CNRS, Paris, 75015 France; 2 Sorbonne Université, IFD, Paris, 75005 France; 3 Mouse Genetics, Institut Pasteur, Paris, 75015 France; 4 Experimental Neuropathology, Institut Pasteur, Paris, 75015 France; 5 Sorbonne Université, INSERM, Physiopathologie des Maladies Génétiques d’Expression Pédiatrique, APHP, Hôpital Armand Trousseau, UF de Génétique Moléculaire, Paris, 75012 France; 6 Lymphopoiesis, Institut Pasteur, U668, INSERM, Paris, 75015 France; 7 Structural Virology, Institut Pasteur, Paris, 75015 France; 8 Laboratório de Imunomodulação e Protozoologia, Instituto Oswaldo Cruz, Fiocruz, Rio de Janeiro Brasil

**Keywords:** Genetic predisposition to disease, Viral host response

## Abstract

Infection of mice with Rift Valley fever virus (RVFV) reproduces major pathological features of severe human disease, notably the early-onset hepatitis and delayed-onset encephalitis. We previously reported that the *Rvfs2* locus from the susceptible MBT/Pas strain reduces survival time after RVFV infection. Here, we used BALB/cByJ (BALB) mice congenic for *Rvfs2* (C.MBT-*Rvfs2*) to investigate the pathophysiological mechanisms impacted by *Rvfs2*. Clinical, biochemical and histopathological features indicated similar liver damage in BALB and C.MBT-*Rvfs2* mice until day 5 after infection. However, while C.MBT-*Rvfs2* mice succumbed from acute liver injury, most BALB mice recovered and died later of encephalitis. Hepatocytes of BALB infected liver proliferated actively on day 6, promoting organ regeneration and recovery from liver damage. By comparison with C.MBT-*Rvfs2*, BALB mice had up to 100-fold lower production of infectious virions in the peripheral blood and liver, strongly decreased RVFV protein in liver and reduced viral replication in primary cultured hepatocytes, suggesting that the BALB *Rvfs2* haplotype limits RVFV pathogenicity through decreased virus replication. Moreover, bone marrow chimera experiments showed that both hematopoietic and non-hematopoietic cells are required for the protective effect of the BALB *Rvfs2* haplotype. Altogether, these results indicate that *Rvfs2* controls critical events which allow survival to RVFV-induced hepatitis.

## Introduction

Rift Valley fever (RVF) is a mosquito-borne viral disease with potential to generate a public health emergency^1^. In humans, infection leads to a variety of clinical manifestations that range from a febrile influenza-like illness with nausea, vomiting and abdominal pain to hepatitis with fatal hemorrhagic fever, encephalitis, retinitis and renal failure^2–5^. In ruminant species, a wide variation in susceptibility to RVF disease is observed among different individuals. Some infected animals suffer from unapparent or moderate febrile reactions^6^ while others develop high fevers with severe liver and renal failure which may lead to death especially in young animals^7,8^. Infected sheep show a high incidence of necrotizing hepatitis, spleen necrosis and renal tubule injury^8^. Sequence analysis of RVF virus (RVFV) strains collected during the 1977–1979 Egyptian outbreak has shown that, although all virus isolates carried virtually identical genotypes, remarkable differences were observed in pathogenesis across human and animal populations^9^. These findings suggest that the different pathogenic phenotypes were not linked to specific mutations in the viral genome but could rather be caused by variations in dose and route of virus exposure and by host-related factors including age, sex, overall immune response, nutritional status and genetic variants.

Careful control of experimental conditions of infection in rodent models has allowed establishing host genetic factors as important determinants in RVF disease severity. The infection of laboratory rodents mimics several features of RVFV-induced pathology in humans, including necrotizing hepatitis and meningoencephalitis^10,11^. The first rat models consisted of the Wistar-Furth (WF) inbred strain which is highly susceptible to the hepatitis induced by subcutaneous inoculation with RVFV, while the Lewis (LEW) strain is largely resistant^10,12^. Inhalation exposure to RVFV confirmed the extreme susceptibility of the WF strain to RVFV-induced hepatitis^13^. The segregation analysis of the RVFV susceptible phenotype in LEW and WF backcrosses suggested a simple Mendelian dominant control^12^. A WF.LEW congenic strain resistant to the fatal hepatitis was created by repeated backcrosses from the resistant LEW to the WF susceptible genetic background^14^. A single region on rat chromosome (Chr) 3 was shown to significantly increase the survival rate of animals carrying the LEW haplotype^15^ but the gene accounting for this improved resistance has yet to be identified.

Mouse inbred strains also exhibit differences in their susceptibility to an infection with RVFV. In one study, the subcutaneous infection of BALB/c mice with 10^3^ plaque-forming units (PFU) of the ZH501 RVFV strain led to an extensive infection of the liver^11^. The resulting liver disease accounted for the death of most animals between days 3 and 6 post infection (p.i.). Mice that survived this early liver disease later developed encephalitis and died around day 8 p.i.^11^. In another study, C57BL/6 mice appeared more susceptible than BALB/c mice under similar experimental conditions and succumbed to acute liver disease within 4 days^16^. We have tested the susceptibility of additional strains derived from various *Mus musculus* subspecies trapped in the wild. The most severely affected strain within this collection, MBT/Pas (MBT), developed very early onset RVF disease. After intraperitoneal infection with 10^2^ PFU of virulent RVFV strain, either Egyptian ZH548 or Kenya 98, MBT mice died more rapidly than BALB/cByJ (BALB thereafter) or C57BL/6 J mice^17^. It is worth noting that MBT mice are susceptible to RVFV but resistant to several other viruses^17^, suggesting that the susceptibility to RVFV exhibited by MBT mice is not attributable to generalized immunodeficiency. In flow cytometry studies, we have recently shown that MBT mice displayed several immunological alterations after RVFV infection including low levels of leukocytes that expressed type I IFN receptor subunit 1 in the blood, spleen and liver, delayed leukocyte activation and decreased percentage of IFN-γ-producing leukocytes in the blood^18^. Furthermore, these mice failed to prevent high viremia and viral antigen loads in the blood, spleen, and liver. We also showed that, in MBT mice, RVF susceptibility is under complex polygenic control and we identified three genomic intervals on Chr 2, 11 and 5 affecting survival time after RVFV infection. Each of these MBT-derived intervals, designated Rift Valley fever susceptibility 1 (*Rvfs1*), *Rvfs2* and *Rvfs3* respectively, conferred reduced survival time in C.MBT congenic strains in which these intervals had been transferred onto the less susceptible BALB genetic background^19^. The pathogenic mechanisms for the early death induced by RVFV in the C.MBT congenic strains are currently unknown.

**Figure 1:**
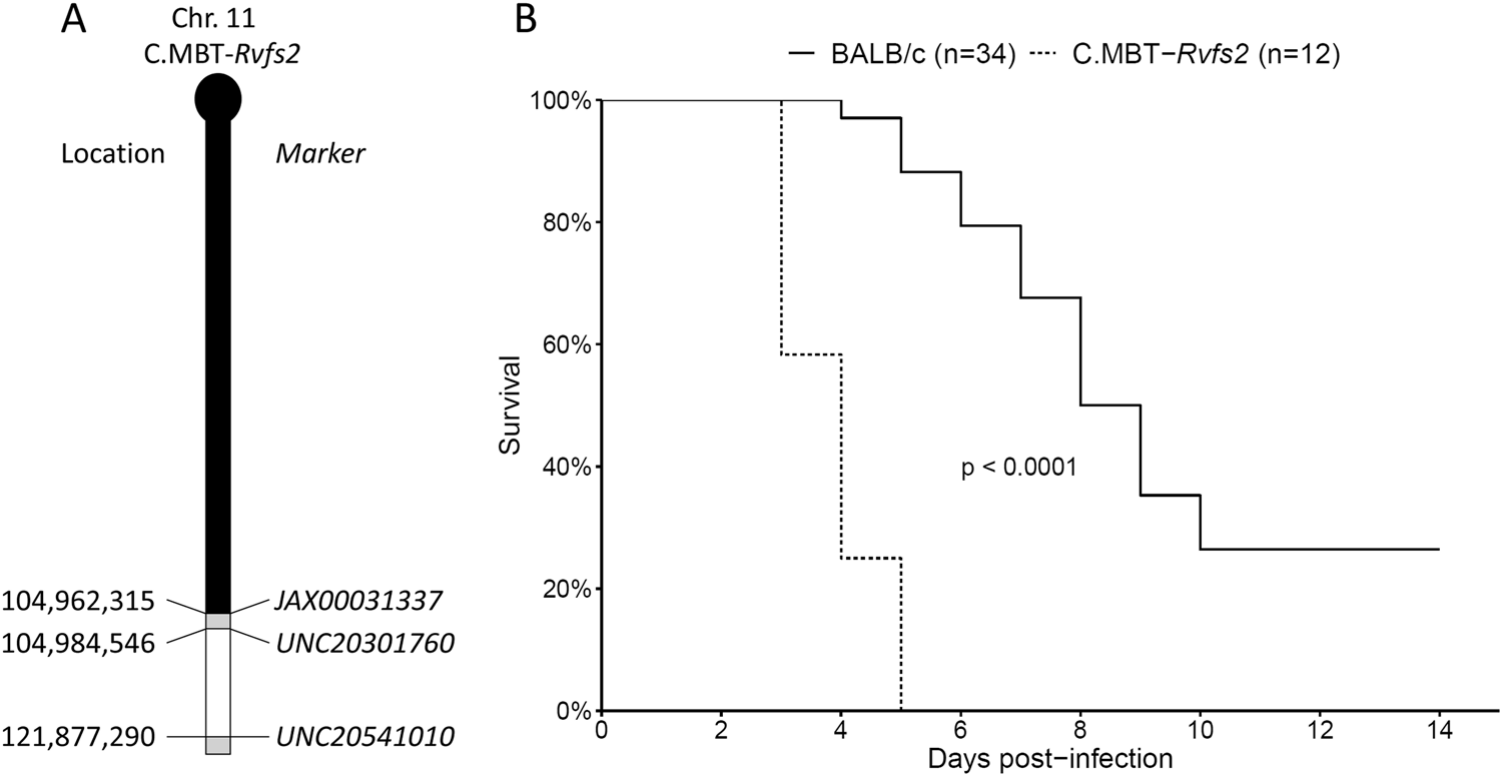
Representation of the MBT-derived *Rvfs2* region in the congenic C.MBT-*Rvfs2* strain and its effect on mouse survival. (**A**) Haplotype structure of the congenic segment of Chr 11 in C.MBT-*Rvfs2* strain. The MBT-derived segment is depicted in white on the BALB/cByJ Chr 11 background (black). Regions of unknown genotype are depicted in grey. Markers are SNPs from the GigaMUGA array (see Table S2 from^37^) and position are given in bp from mouse Genome Build 38 (corrected from^19^). (**B**) Survival curves of C.MBT-*Rvfs2* and BALB male mice infected with 100 PFU IP (Mantel-Cox’s Logrank test; p < 0.0001).

In this study, we investigated the phenotypic features associated with morbidity in BALB mice and in C.MBT-*Rvfs2* mice which carry a ≈ 17 Mb segment of Chr 11 region of the MBT strain encompassing the *Rvfs2* interval in the BALB background (Fig. 1A). We focused our investigations on male mice which exhibit slightly higher susceptibility to RVFV infection^19^. The study of clinical, biochemical and virological parameters, as well as histopathological features of the RVF disease showed that mice from both BALB and C.MBT-*Rvfs2* inbred strains exhibited hepatic disease. The first clinical signs of disease were detected on the third day of infection in both strains. However, while C.MBT-*Rvfs2* mice began to die on day 3 of acute liver disease, most BALB mice recovered and died three to nine days later of encephalitis. Since MBT-derived *Rvfs2* haplotype was associated with increased viral load in the liver and higher viral replication rate in primary cultured hepatocytes, we suggest that the BALB *Rvfs2* haplotype limits RVFV pathogenicity through decreased virus replication. Since our previous work has revealed immune-related defects in MBT mice in response to RVFV infection^18^, we assessed the contributions of hematopoietic and non-hematopoietic cells in the effects of BALB and MBT *Rvfs2* haplotypes using chimeric mice produced by crosswise transplantations of bone-marrow cells after total body irradiation between BALB and C.MBT-*Rvfs2* mice. We showed that both hematopoietic and non-hematopoietic cells are required for the capacity of BALB mice to survive RVFV-induced liver damages.

## Results

### **BALB and C.MBT-*Rvfs2*mice show evidence of liver disease at the early stage of infection**

The challenge of C.MBT-*Rvfs2* mice with 10^2^ PFU of the ZH548 RVFV strain showed that *Rvfs2* has a strong effect on susceptibility to RVFV. All C.MBT-*Rvfs2* mice died between days 3 and 5 p.i., whereas 50% of BALB mice survived for 8 days or more, and 26% (9/34) survived (Fig. 1B, Mantel-Cox’s Logrank test, p < 0.0001).

**Figure 2:**
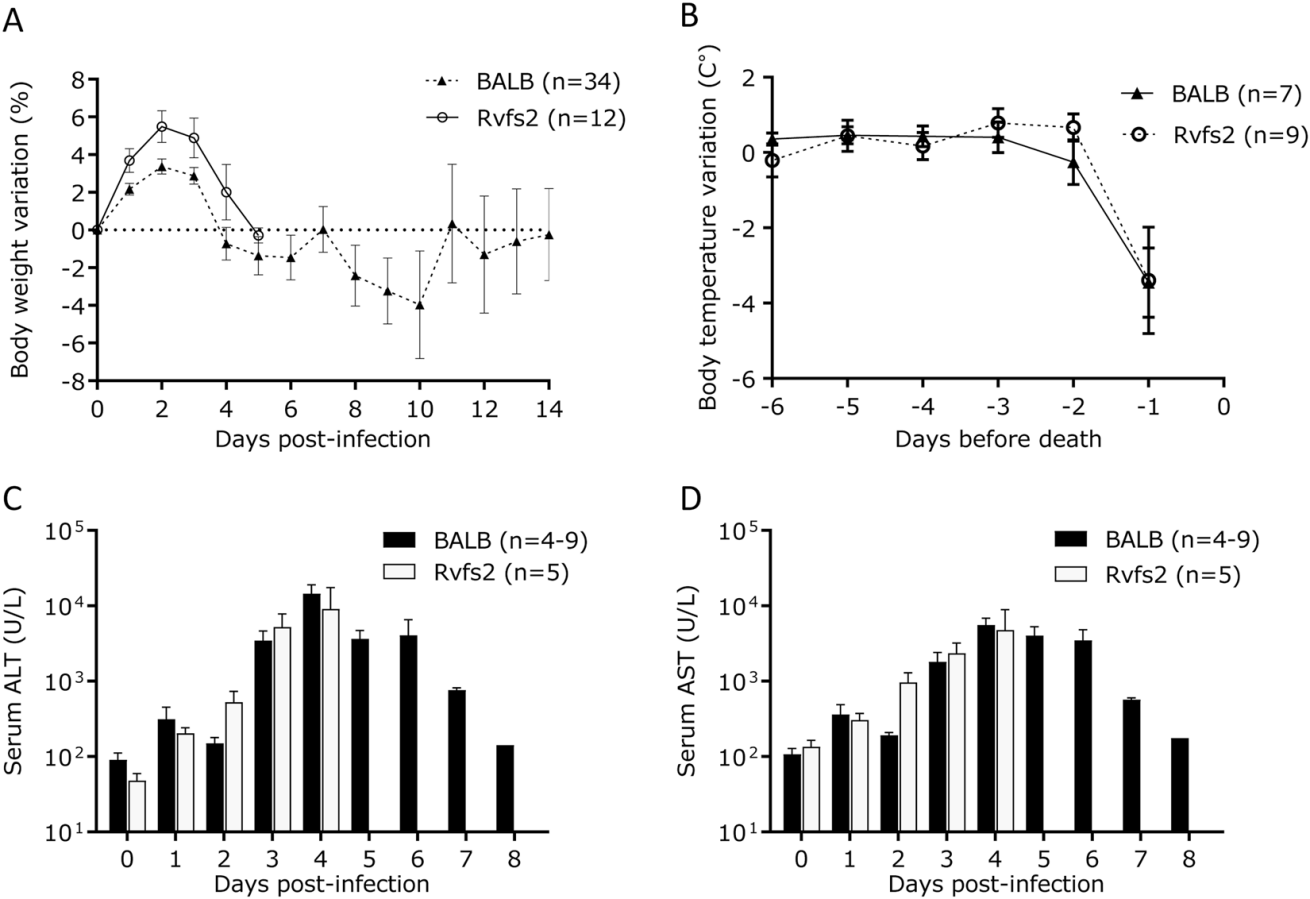
Clinical traits and biochemical parameters of RVFV-infected C.MBT-*Rvfs2* and BALB mice. (**A**) Body weight variation in C.MBT-*Rvfs2* (Rvfs2) and BALB mice after infection with RVFV ZH548 (mean ± SEM). (**B**) Body temperature variation in C.MBT-*Rvfs2* and BALB mice during the days preceding the death (mean ± SEM). No difference was observed between the two strains (two-way ANOVA, p = 0.69). (**C,D**) Alanine aminotransferase (ALT) (**C**), and aspartate aminotransferase (AST) (D) levels in the serum of C.MBT-*Rvfs2* and BALB mice. By day 5 p.i., all C.MBT-*Rvfs2* mice had died. Data are means ± SEM for N = 4-9 mice per day, except for day 8 in BALB mice where N = 1.

Clinical signs of disease in RVFV-infected C.MBT-*Rvfs2* mice were monitored daily in comparison with BALB mice. C.MBT-*Rvfs2* mice developed signs of an acute disease with ruffled fur, hunched appearance and lethargy as early as days 3 and 4 p.i. In contrast, most RVFV-infected BALB mice exhibited the first symptoms of disease later, from day 6 p.i. BALB mice showed different degrees of clinical signs, including ascending paralysis, ataxia, head-tilt and circling behavior. Body weight and body temperature were also measured daily in RVFV-infected mice. Body weight variations were similar in both strains during the first 5 days p.i., with an increase during the first two days and a decrease between days 2 and 5 p.i. when all C.MBT-*Rvfs2* mice died (Fig. 2A). In BALB mice, body weight remained stable between days 4 and 7 p.i. and decreased again between days 7 and 10 p.i. Temperature measurements indicated that neither BALB nor C.MBT-*Rvfs2* mice developed hyperthermia during the course of infection. In the mice that died of RVFV infection, a significant drop in body temperature was only observed one day before death in both inbred strains, regardless of the time of death (Fig. 2B). Overall, no differences in body temperature between BALB and congenic mice were found (two-way ANOVA, P(strain effect) = 0.69).

Liver enzymes alanine aminotransferase (ALT) and aspartate transaminase (AST) measured during the disease course peaked on day 4 p.i. in both BALB and C.MBT-*Rvfs2* mice (Fig. 2C,D), indicating hepatocyte damage. After day 5 p.i., AST and ALT serum levels decreased slowly in infected BALB mice and returned to normal levels on day 8 p.i., suggesting recovery from the liver disease. Altogether the development of the RVF disease in the first 3–4 days was similar in both inbred strains.

### **BALB and C.MBT-*Rvfs2*mice exhibit liver damage at days 3 and 4 p.i**

**Figure 3:**
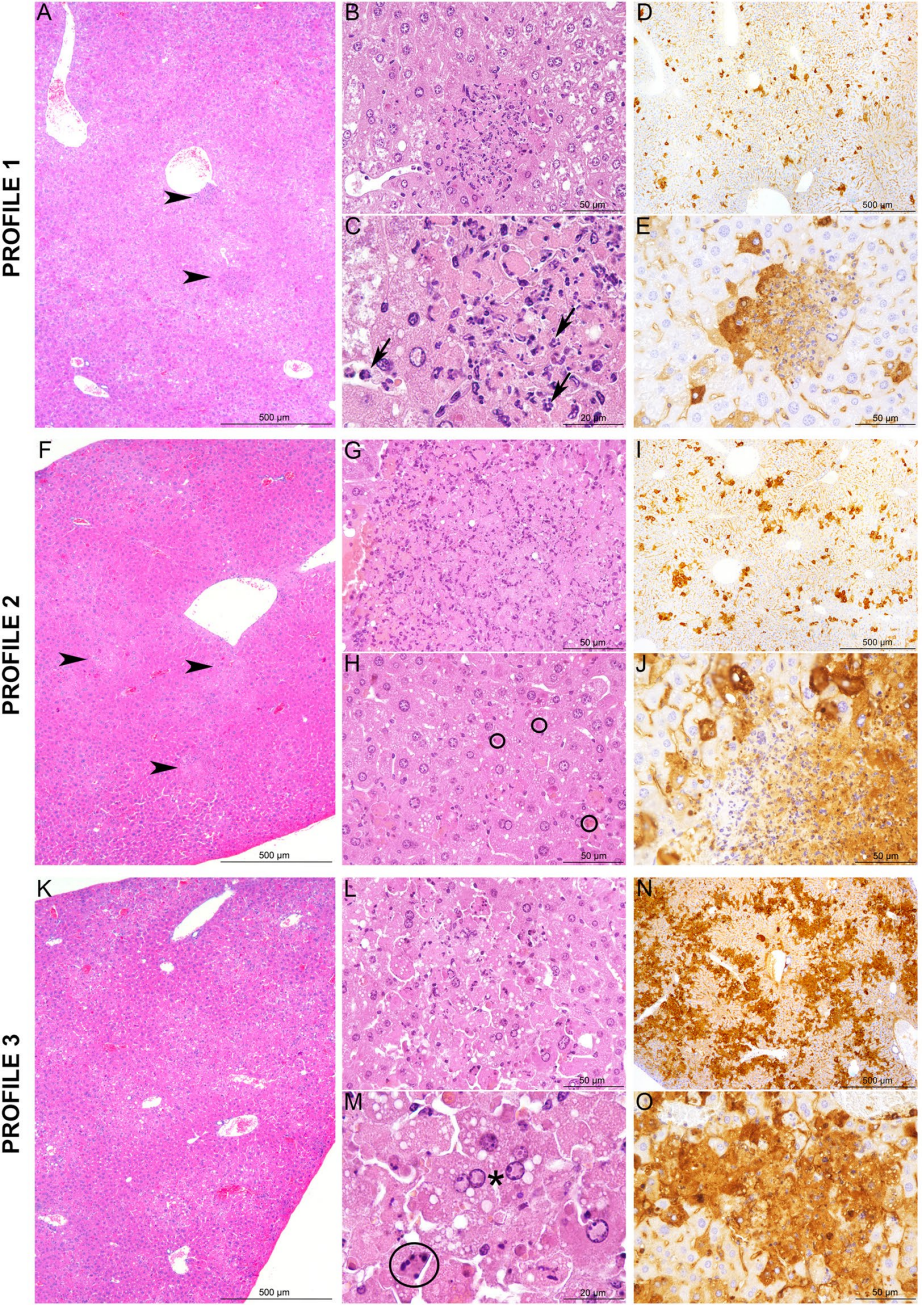
Histopathology and immunohistochemistry analyses of liver from BALB and C.MBT-*Rvfs2* mice on day 3 p.i. Three distinct histological profiles were found in 10 BALB and 8 C.MBT-*Rvfs2* infected mice. Profile 1: (**A**) Randomly distributed, multifocal inflammatory lesions (arrowheads) with (**B**,**C**) small well-delimited foci of necrotic/apoptotic hepatocytes associated with neutrophil infiltration (**C**, black arrows). (**D**,**E**) Small clusters of RVFV N protein-positive hepatocytes recognized by immunohistochemistry. Profile 2: (**F**) Multifocal inflammatory lesions randomly distributed in the liver (arrowheads) with (**G**,**H**) more extensive and severe foci of necrotic/apoptotic hepatocytes than in Profile 1 (H, black circles: apoptotic bodies). (**I**,**J**) Slightly larger clusters of N-positive hepatocytes observed after immunohistochemistry. Profile 3: (**K**–**M**) Massive necrosis/apoptosis of hepatocytes (M black circle: apoptotic body; black star: intranuclear viral inclusion), (**N**,**O**) with a strong and diffuse immunohistochemistry staining for RVFV N protein in the parenchyma. None of these lesions were observed in the liver of uninfected BALB and C.MBT-*Rvfs2* mice. A, B, C, F, G, H, K, L, M: Hematoxylin and eosin staining; D, E, I, J, N, O: Immunohistochemistry for RVFV N protein.

We studied the tissue damage caused by RVFV in the liver of infected BALB (N = 10) and C.MBT-*Rvfs2* (N = 8) mice euthanized on day 3 p.i. at a stage where most BALB mice were asymptomatic while C.MBT-*Rvfs2* mice had developed mild to moderate clinical symptoms (reduced movement, ruffled fur). Histopathological analyses of the liver, focusing on three prominent histological features (distribution of the lesions, severity of the inflammatory reaction, presence of focal or extensive necrosis/apoptosis), revealed three different lesion profiles of increasing severity in both mouse genotypes (Fig. 3). Five out of 10 BALB and 5/8 C.MBT-*Rvfs2* mice exhibited mild, multifocal and well demarcated lesions defined as Profile 1. Liver lesions in these mice were characterized by hepatocyte cell death associated with small inflammatory infiltrates containing fragmented neutrophils (Fig. 3A–C). Immunohistochemical (IHC) labeling directed against the viral N protein, used to identify infected cells, revealed small multifocal lesions (less than 100 µm in diameter) of infected hepatocytes (Fig. 3D,E). Profile 2 was observed in 3/10 BALB and 2/8 C.MBT-*Rvfs2* mice. This profile was also characterized by multifocal lesions with hepatocyte cell death. However, lesions were more severe and extensive (Fig. 3F–H). IHC analyses detected a stronger signal with slightly larger foci of infected hepatocytes (Fig. 3I,J). A third profile was observed in 2/10 BALB and 1/8 C.MBT-*Rvfs2* mice. Liver sections categorized as Profile 3 displayed severe and extensive tissue damage with minimal inflammation. Lesions were characterized by acute and massive cell death of hepatocytes, numerous viral inclusion bodies in the nuclei of cells (Fig. 3K–M), and an extensive positive immunolabeling of hepatocytes for the viral N protein (Fig. 3N,O). None of these lesions were observed in liver sections of uninfected BALB and C.MBT-*Rvfs2* mice. Collectively, these results indicated that BALB and C.MBT-*Rvfs2* mice experienced similar liver conditions on day 3 p.i. with the same range of histological lesions, from mild to severe, up to extensive destruction of the liver parenchyma. Overall, non-quantitative IHC indicated, in each profile, similar densities of RVFV-infected liver cells in both strains.

We then examined the liver of C.MBT-*Rvfs2* mice which presented more severe symptoms (prostration, tremor) and had reached the humane endpoint defined in the protocol as requiring euthanasia. Notably, this aggravation of disease severity was rarely observed in BALB mice. Five such moribund congenic mice were euthanized on days 3 (N = 1), 4 (N = 2) and 4.5 (N = 2) p.i. All five livers displayed severe and non-inflammatory lesions, characterized by massive and acute cell death, consistent with Profile 3 but with extensive lesions. This result suggests that, in C.MBT-*Rvfs2* mice, the disease progressed from mild inflammation to non-inflammatory liver lesions and continued to aggravate, resulting in extensive tissue damage. Lesions observed in the liver of moribund C.MBT-*Rvfs2* mice were sufficient to alter liver function, and could have led to the rapid death of infected animals.

### Infected BALB mice survive the early-onset liver disease, but succumb later to encephalitis

**Figure 4:**
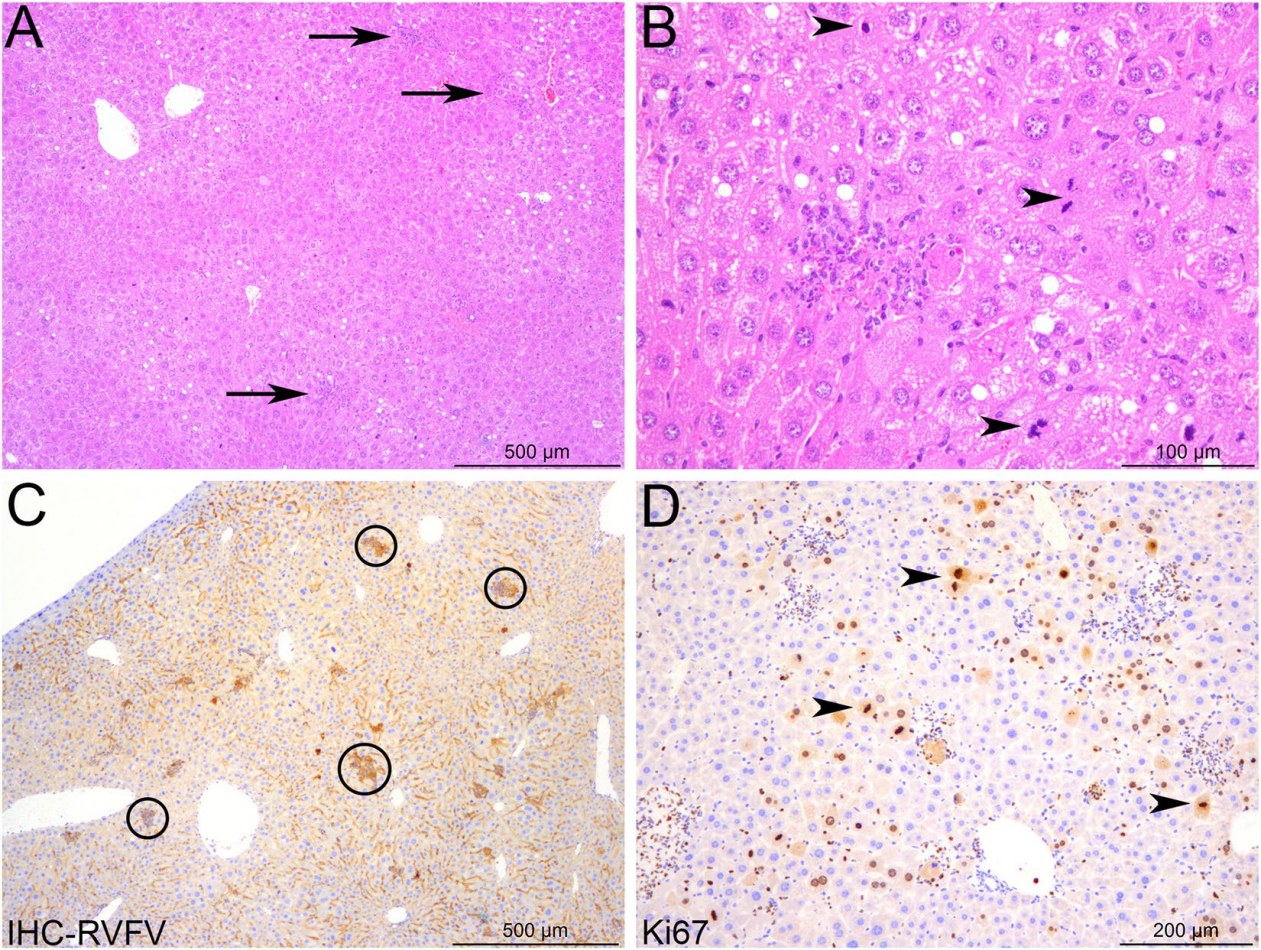
Hepatocyte proliferation and liver regeneration in BALB mice recovering from RVFV-induced liver disease. Liver sections of four BALB mice were examined at day 6 post-infection. (**A**) Rare and randomly distributed lesions in the liver parenchyma are observed (arrows). (**B**) Small infiltrates of inflammatory cells (probably neutrophils and Kupffer cells) associated with focal hepatocyte destruction may be observed in the lesions. Increased mitotic activity is seen among hepatocytes (arrowheads). (**C**) Immunohistochemistry for RVFV N protein reveals a weak signal, only detected in the small foci identified in hematoxylin and eosin-stained sections (black circles). (**D**) Immunohistochemistry for Ki67 highlights a marked, diffuse proliferation of the hepatocytes (arrowheads). (**A**,**B**) Hematoxylin and eosin staining; (**C**) Immunohistochemistry for RVFV N protein; (**D**) Immunohistochemistry for Ki67.

**Figure 5:**
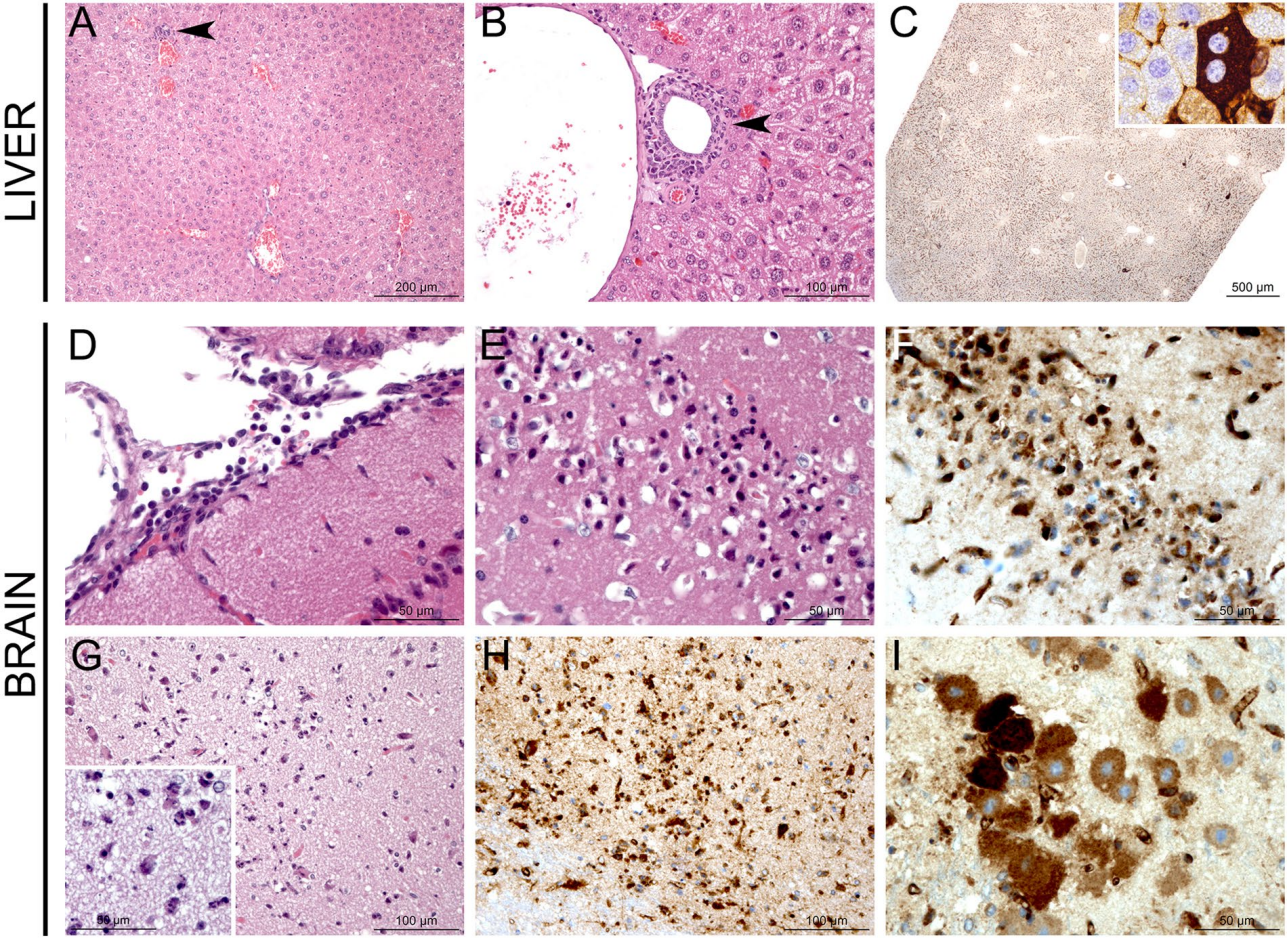
Histopathology and immunohistochemistry analyses of liver and brain from moribund BALB mice. (**A**–**C**) Liver from a moribund BALB mouse on day 8.5 p.i. displays minimal multifocal inflammatory lesions either randomly distributed in the liver parenchyma (arrowhead) (**A**) or centered on portal tracts, mostly around bile ducts (arrowhead) (**B**). Rare RVFV-infected cells are indicated by IHC with antibodies against RVFV N protein (**C**). (**D**–**I**) Brains from moribund BALB mice on days 7 to 9 p.i. display different inflammatory and apoptotic/necrotic lesions: subacute leptomeningitis characterized by infiltration of leptomeninges by lymphocytes, plasma cells and neutrophils (**D**), laminar apoptosis/necrosis of neurons in the cortical outer granular layer (**E**) with RVFV N protein-positive neurons (**F**), necrotic/apoptotic foci in different locations of the cerebral grey matter with gliosis (**G**) and infiltration of neutrophils (inset), and strong signal for RVFV N protein (**H**-**I**). Histology and immunohistochemistry results shown are representative of experiments performed on at least 4 animals. A, B, D, E, G: Hematoxylin and eosin staining; C, F, H, I: Immunohistochemistry for RVFV N protein.

The gradual decrease of liver transaminases between days 5 and 8 post-infection in BALB mice suggested hepatic tissue regeneration. On day 6 p.i., examination of histopathological changes in livers of moribund BALB mice (N = 4) revealed only minimal lesions (Fig. 4A,B) and few RVFV N-positive hepatocytes were detected (Fig. 4C). An increased number of mitotic cells as well as a strong and diffuse expression of Ki67 confirmed the proliferation of hepatocytes (Fig. 4D). By day 8 p.i., minimal to mild, subacute to chronic inflammatory lesions were scattered in the liver parenchyma or centered on portal tracts and consisted of small infiltrates of lymphocytes, plasma cells and macrophages (Fig. 5A,B). Very few hepatocytes were labeled positively for the RVFV N protein, confirming an efficient viral clearance in the hepatic tissue (Fig. 5C). Since BALB mice exhibited clinical neurological signs, we investigated their brain for infection-related lesions. Histopathological lesions were visible in the brain of moribund BALB mice. The virus targeted different brain anatomic structures in each individual mouse, and no pathognomonic lesion profile could be defined (Fig. 5D–I). We detected (i) subacute leptomeningitis with multifocal infiltration of the leptomeninges by lymphocytes, plasma cells, and neutrophils (Fig. 5D), and (ii) cell death foci in different locations of the cerebral grey matter, e.g. the outer granular layer or different brain nuclei (Fig. 5E–G). In these foci, shrinkage of neurons, gliosis, infiltration of neutrophils and strong RVFV N protein immunolabeling of neurons were observed (Fig. 5H,I). These lesions were likely the cause of the neurological symptoms and eventual death in BALB mice.

### **Elevated viral burden in the blood and liver of C.MBT-*Rvfs2*mice**

**Figure 6:**
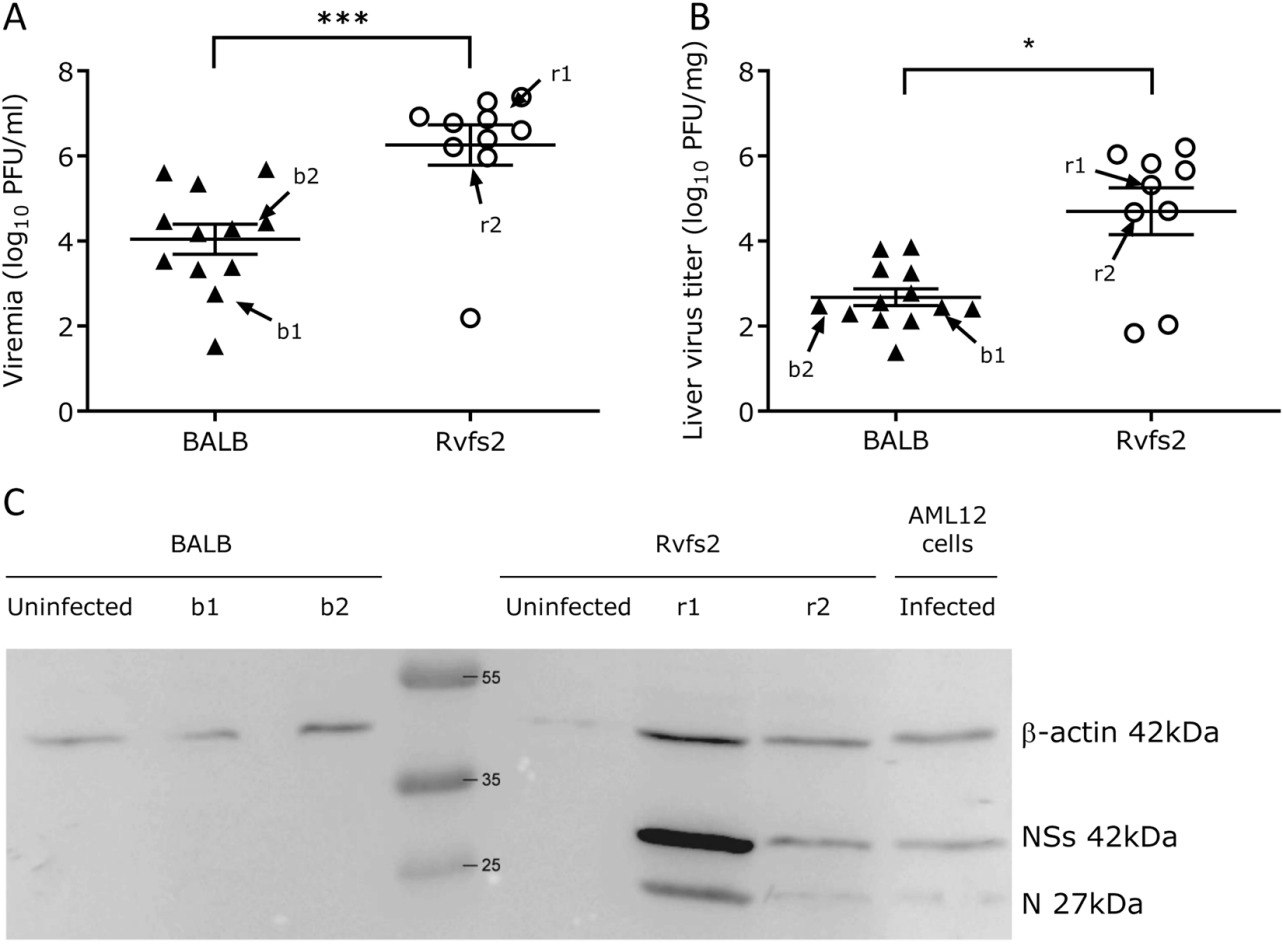
Production of viral particles and viral proteins in BALB and C.MBT-*Rvfs2* mice on day 3 p.i. (**A**) Viremia in RVFV-infected C.MBT-*Rvfs2* (Rvfs2) (N = 10) and BALB (N = 12) mice. (**B**) Viral titers in liver from C.MBT-*Rvfs2* (N = 9) and BALB (N = 13) mice (**C**) Western blotting analysis of the liver from BALB and C.MBT-*Rvfs2* (Rvfs2) uninfected (N = 1) and infected mice on day 3 p.i. (N = 2, from the groups of mice analyzed in A and B and identified as b1, b2 for BALB and r1, r2 for Rvfs2). RVFV-infected AML12 cells were included as a positive control. Proteins were analyzed with antibodies against NSs and N viral proteins, and beta-actin. The molecular weight and positions of the marker bands (middle lane), and NSs, N and β-actin proteins (right lane) are indicated.

The titers of infectious viral particles on day 3 p.i. were about 80- and 100-fold higher in the C.MBT-*Rvfs2* blood and liver, respectively, compared with those found in BALB mice (Mann Whitney-U test, p < 0.001 and p = 0.016, respectively; Fig. 6A,B). Semi-quantitative protein analysis of liver extracts at day 3 p.i. by Western blot indicated that high levels of N nucleocapsid and NSs nonstructural viral proteins were found in the liver of C.MBT-*Rvfs2*, while both viral proteins were undetectable in BALB liver (Fig. 6C) despite the same proportions of RVFV-infected cells in the two strains revealed by IHC (Fig. 3). Altogether, these results indicated that, compared to C.MBT-*Rvfs2* mice, BALB mice allow lesser replication of RVFV, thus limiting the production and dissemination of the virus systemically.

### Increased viral replication in C.MBT-*Rvfs2*-derived cultured primary hepatocytes

**Figure 7:**
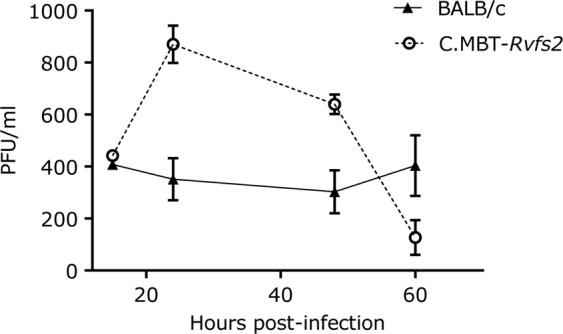
Viral replication in primary cultured hepatocytes from BALB and C.MBT-*Rvfs2* mice. Virus titer measured in the supernatant of primary cultured hepatocytes, at 15, 24, 48 and 60 hr p.i. with RVFV at MOI of 3. Error bars from technical triplicates. Experiment representative of two.

We measured the kinetics of viral production in the culture medium of primary hepatocytes derived from the liver of BALB and C.MBT-*Rvfs2* uninfected mice, over 60 h after infection with RVFV. While the viral titer remained constant at 300–400 PFU/ml in the BALB culture, it peaked in C.MBT-*Rvfs2*-derived hepatocytes at almost 900 PFU/ml 24 h after infection before decreasing at 48 and 60 hours post-infection (Fig. 7).

### **Both hematopoietic and non-hematopoietic cells are required for*Rvfs2*-dependent survival to liver disease**

**Figure 8:**
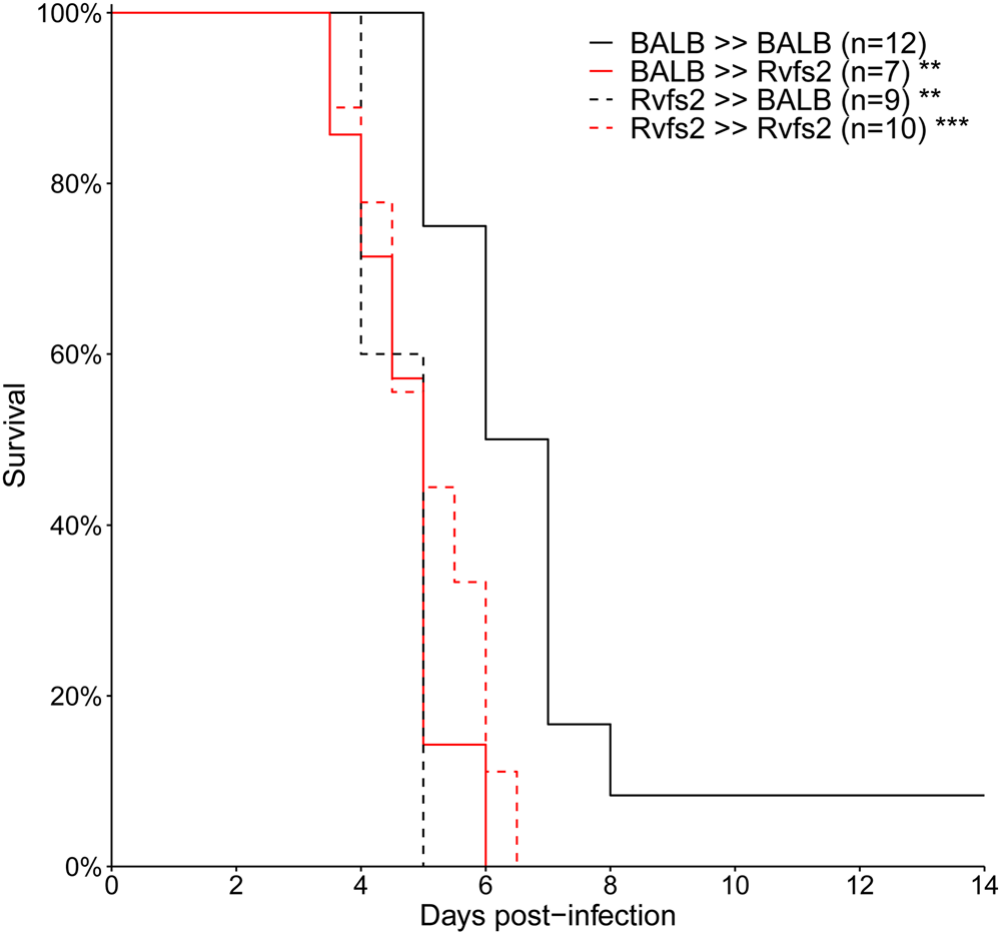
Survival curves of chimeric mice generated by reciprocal transplantation of bone marrow cells. Sub-lethally irradiated C.MBT-*Rvfs2* (Rvfs2, red lines) or BALB (black lines) recipient mice received ~3 × 10^6^ bone marrow cells from either C.MBT-*Rvfs2* (dashed lines) or BALB (solid lines) donor mice on the same day as irradiation. The recipient mice were infected intraperitoneally with 10^2^ PFU RVFV six weeks later. Asterisks refer to the comparison between each group and the BALB → BALB control group (Mandel-Cox’s Logrank test; **p < 0.01, ***p < 0.001).

We infected with RVFV BALB mice reconstituted with C.MBT-*Rvfs2* marrow (C.MBT-*Rvfs2* → BALB chimeras), C.MBT-*Rvfs2* mice reconstituted with BALB marrow (BALB → C.MBT-*Rvfs2* chimeras), as well as controls consisting of irradiated mice reconstituted with isogenic marrow, (BALB → BALB and C.MBT-*Rvfs2* → C.MBT-*Rvfs2* chimeras). As shown in Fig. 8, BALB → BALB chimeras survived significantly longer than C.MBT-*Rvfs2* → C.MBT-*Rvfs2* chimeras (Mantel-Cox’s Logrank test, p = 0.0002), like the non-manipulated BALB and C.MBT-*Rvfs2* strains (Fig. 1B). Interestingly, the survival time was significantly shorter in C.MBT-*Rvfs2* → BALB chimeras compared with BALB → BALB chimeras (Mantel-Cox’s Logrank test, p < 0.01). By contrast, the survival time of BALB → C.MBT-*Rvfs2* chimeras was not increased compared to C.MBT-*Rvfs2* → C.MBT-*Rvfs2* chimeras (Mantel-Cox’s Logrank test, p = 0.78). Therefore, only mice having both hematopoietic and non-hematopoietic compartments from the BALB strain were protected against early fatal hepatitis.

## Discussion

There is considerable variability in the ability of patients and livestock to survive RVF disease. A number of factors, such as the viral strain, the route and dose of viral exposure and the age, sex, nutritional and immune status of the host, can modulate the severity of the disease and contribute to the balance between recovery and death, a complex phenotype that involves multiple systems, organs, tissues, immune cells and cellular pathways, under the influence of multiple genes. The importance of inoculation route and dose and of the age and sex of the host has been experimentally demonstrated in laboratory rodents^12–14,17,20^. Experiments in laboratory rodents have further demonstrated that survival time and survival rate following RVFV infection are influenced by host genetic determinants^12,17^. Our previous studies have shown that, after intraperitoneal injection of 10^2^ PFU of the ZH548 RVFV strain, mice of the inbred MBT mouse strain died within 5 days p.i. with clinical signs of liver disease. By contrast, most BALB mice lived beyond that date and exhibited signs of encephalitis, such as paralysis, ataxia, or head-tilting behavior^17^. Notably, the difference in the days of death between BALB and MBT mice was identical at infectious doses ranging from 10 to 1000 PFU (S1 Table). We demonstrated that susceptibility to RVF in the inbred MBT mouse strain is inherited as a multigenic trait, with sex influencing the severity of the disease. Three RVF susceptibility loci (*Rvfs*) with a moderate effect on the survival time were identified^19^. The introgression by repeated backcrosses of each of these chromosomal regions within the less susceptible genetic background BALB led to congenic mice exhibiting significant reduction in survival time compared to the BALB control groups^19^. Functional studies are needed to unravel the nature and the role of the genes within the *Rvfs* loci in the pathogenesis of RFV. We focused our efforts first on *Rvfs2*, a 17 Mb genomic interval on distal Chr 11, because of its strongest effect.

The pathogenesis induced by the subcutaneous challenge of BALB mice with 10^3^ PFU ZH501 RVFV has been recently characterized in detail^11,21^. Approximately 80% of BALB mice infected in these conditions were reported to have succumbed with severe liver disease between days 3 and 6 p.i., a much higher percentage than the one observed in our study (~20%). Several experimental factors differ between the two studies. Although ZH501 and ZH548 RVFV strains have been isolated in the same hospital during the 1977 Egyptian outbreak, they have distinct passaging history^11^ and exhibit a small percentage of nucleotide differences^9,22,23^. Therefore, we cannot exclude that ZH501 and ZH548 RVFV strains induce distinct survival rates at day 6 p.i. Based on our previous experiments (S1 Table), a lower inoculation dose (10^2^ instead of 10^3^ PFU) is unlikely to be solely responsible for a reduced death rate between days 3 and 6 p.i. in our study. Conversely, the two studies used different routes of infection (SC versus IP) which result in different virus dissemination in tissues and kinetics of death^24^. Finally, this difference in RVFV disease presentation and progression could be due to mouse sex and genetic background since we used males of the BALB/cByJ inbred strain while the other study was performed on female BALB/c mice, without indication of the substrain. Significant differences between BALB/c substrains have been previously reported with other infectious diseases and immune responses^25,26^, emphasizing the importance of accurately specifying the animal strain used is such studies. Whatever the reason for this difference in survival rates, our findings are consistent with the biphasic RVF disease reported by Smith and colleagues^11^ that consists of an acute liver disease followed by a panencephalitis.

Under our conditions, C.MBT-*Rvfs2* mice were highly susceptible to, and died from, the early-onset liver disease, while BALB mice overcame it and died later of encephalitis. Our results suggest that one of the mechanisms underlying the ability of BALB mice to survive liver disease is a reduced viral replication at the cellular level, as shown in primary cultured hepatocytes and by the decreased viral load in the liver, despite similar percentage of infected liver cells as assessed by non-quantitative IHC. Altogether, these findings establish the feasibility and exemplify the value of segregating important sub-phenotypes by transferring a single locus, *Rvfs2*, from the early susceptible to a late susceptible background. Susceptibility to liver disease has also been reported in WF inbred rats after subcutaneous infection with RVFV ZH501^10^. WF rats died by day 2 post inoculation of liver necrosis, whereas LEW rats were resistant to the liver disease but fairly susceptible to the encephalitis^10,14^. This susceptibility to liver necrosis occurred in a similar time frame after respiratory infection in WF rats^13^. The pathogenic mechanisms that trigger the susceptibility of WF rats and C.MBT-*Rvfs2* mice to RVF hepatic disease may be similar. Indeed, it has been reported that susceptible WF rats had much higher blood viral titers than resistant LEW rats at day 2 p.i^14^., in line with the higher viral production in hepatocytes from WF rats compared with LEW rats^27^. These data suggest that the rat susceptibility locus also controls the production of RVFV. Recently, a major gene for the susceptibility has been mapped within a region on rat Chr 3^15^. This rat region has homology with mouse Chr 2, indicating that the rat susceptibility locus and *Rvfs2* which maps on mouse Chr 11 do not point at the same gene(s). Therefore, the genetic variations captured in WF rats and MBT mice are different, which makes both rodent models equally interesting and important.

In principle, the RVFV-infected host can protect itself from fatal liver disease through two non-mutually exclusive mechanism^28,29^. Resistance refers to the capacity of reducing viral burden once the infection is established, while tolerance reduces the negative impact of the infection on host fitness (i.e. the same viral titer resulting in less alterations of physiological functions). Our results indicate that the ability of BALB mice to survive the early-onset liver disease can be primarily attributed to lower levels of RVFV in the blood and liver and lesser viral replication in hepatocytes compared with C.MBT-*Rvfs2* mice. Whether protection from liver disease is only due to higher resistance to viral replication or also to increased tolerance to RVFV-induced cell damages remains to be determined.

Reciprocal transplantation of bone-marrow cells between BALB and C.MBT-*Rvfs2* mice was used to analyze the contribution of hematopoietic and non-hematopoietic cells to the protection against early fatal hepatitis induced by the BALB *Rvfs2* haplotype. This experimental approach has previously been used for other pathologies such as auto-immune diabetes^30^. The earlier death of C.MBT-*Rvfs2* → BALB chimeras compared with BALB → BALB chimeras indicates either that C.MBT-*Rvfs2* hematopoietic cells are able to confer to BALB mice susceptibility to RVFV-induced fatal hepatitis or, reciprocally, that BALB hematopoietic cells are required to confer the resistance observed in BALB mice. The observation that the survival curves of BALB → C.MBT-*Rvfs2* and C.MBT-*Rvfs2* → C.MBT-*Rvfs2* chimeras were not different indicates that BALB hematopoietic cells alone are not sufficient to confer the protection against early fatal hepatitis observed in BALB mice. Altogether, our results demonstrate an absolute requirement of both BALB hematopoietic and BALB non-hematopoietic cells for the resistance induced by the BALB *Rvfs2* haplotype. Understanding how *Rvfs2* specifically impacts the functions of hematopoietic cells in fighting RVFV infection will require further studies. It is possible that, as for other cell types such as hepatocytes, the BALB *Rvfs2* haplotype reduces RVFV multiplication in bone-marrow-derived cells. We have previously shown that viral replication was higher in mouse embryonic fibroblasts derived from MBT/Pas than from BALB^17^. On the other hand, our previous work has unraveled a delayed and partial type I interferon response in MBT mice^17^ as well as other immunological differences^18^ which could contribute to the susceptibility associated with the MBT *Rvfs2* haplotype. While these two previous studies compared two genetically distant inbred strains (MBT and BALB), we have focused in this report on two strains which differ only for a 17 Mb genomic interval and we provide insight into the role of the *Rvfs2* locus in the ability to survive the RVF-induced liver disease. Considering that this locus contains over 250 protein-coding genes, it is possible that its overall effect results from the combined action of linked genes involved in distinct pathways or molecular or cellular processes. Reducing the size of the *Rvfs2* interval will pave the way for the identification of causal variants and may uncover new mechanisms of resistance to RVFV.

## Materials and Methods

### Ethics statement

Experiments on mice were conducted according to the French and European regulations on care and protection of laboratory animals (EC Directive 2010/63/UE and French Law 2013–118 issued on February 1, 2013). All experimental protocols were approved by the Institut Pasteur Ethics Committee (under #2013-0127, 2016-0013 and dap160063) and authorized by the French Ministry of Research (under #02301, 06463 and 14646, respectively).

### Mice

C.MBT-(JAX00031337-UNC20541010) congenic mice, designated herein as C.MBT-*Rvfs2*, carry a segment of Chr 11 from the MBT/Pas (MBT) inbred strain extending between positions 104,962,315 and 121,877,290 in assembly mapping GRCm38, in a BALB/cByJ (BALB) inbred genetic background (Fig. 1A)^19^. This region contains approximately 278 protein-coding genes (http://www.informatics.jax.org). C.MBT-*Rvfs2* and BALB mice were bred under specific pathogen-free conditions at the Institut Pasteur.

### Virus production and mouse infection

The RVFV strain ZH548, isolated from a male patient with the acute febrile illness at Zagazig fever hospital, Egypt^23,31^ (obtained from Centre National de Référence des Fièvres Hémorragiques Virales, Institut Pasteur, Lyon, France), was used for all infection studies. All experiments that involved virulent RVFV were performed in the biosafety level 3 (BSL3) facilities of the Institut Pasteur, and carried out in compliance with the recommendations of the Institut Pasteur Biosafety Committee (N° 14.320). Stocks of RVFV ZH548 were titrated by plaque assay on monolayers of Vero E6 cells^32^. Infections were carried out on 9 to 13 weeks old male mice, in BSL-3 isolators. Mice were infected intraperitoneally with 10^2^ PFU of RVFV strain ZH548 except for the experiment shown in the S1Table (10 to 1000 PFU). Clinical disease scores and mortality were monitored daily for 14 days following infection. Moribund animals were euthanized. Animals that survived were euthanized on the last day of the monitoring period.

### Clinical evaluation

Implantable Programmable Temperature Transponders (IPTT-300) (Bio Medic Data Systems, Inc., Seaford, DEL, USA) were injected subcutaneously into mice one week prior to challenge with RVFV ZH548, and body temperature was monitored daily. Body weight of ZH548-infected mice was measured daily throughout the course of the experiment to evaluate the daily weight loss. Alanine aminotransferase (ALT) and aspartate aminotransferase (AST) levels were measured using IDEXX diagnostic panels analyzed on a VetTest chemistry analyzer (IDEXX laboratories, Westbrook, ME, USA) on ZH548-infected mice and uninfected controls. Blood was collected on heparin-lithium and RVFV was inactivated by incubating plasma for 20 minutes with 0.5% sodium deoxycholate and 1% Triton X-100 (Sigma-Aldrich, Saint-Quentin Fallavier, France).

### Viral titer, viral RNA load, and expression of N and NSs viral proteins

Groups of infected BALB and C.MBT-*Rvfs2* mice were euthanized on day 3 p.i. Blood was collected by cardiac puncture. The left lateral lobe of the liver was harvested after perfusion from the portal to the cava vein with saline to remove blood-associated RVFV from the tissues. Infectious titers were measured in sera samples and liver homogenates by plaque assay on monolayers of Vero E6 cells^32^.

The expression of N and NSs viral proteins was studied by Western blot analysis. Total proteins were extracted from liver samples of two mice used above for viral titration (noted b1, b2, r1 and r2 on Fig. 6). Protein quantification was done using Micro BCA Protein Assay kit (ThermoFisher Scientific, Waltham, MA). Ten μg of total protein from a cell lysate from AML12 hepatocytes infected with RVFV at an MOI of 3 were used as a positive control. Forty micrograms of total proteins extracted from liver samples and resuspended in Laemmli buffer were run on 14% SDS-polyacrylamide gel and transferred onto nitrocellulose membranes (Amersham, Velizy-Villacoulay, France). Membranes were blocked with a solution of 5% milk (low fat) in PBS containing 0.05% of Tween 20 also used to dilute antibodies (Ab). Proteins were detected by using a rabbit polyclonal Ab raised against a recombinant N protein produced in the baculovirus system, a mouse polyclonal Ab raised against the entire NSs protein^33,34^, or a monoclonal anti-β-actin antibody (A5441, Sigma-Aldrich, Saint-Quentin Fallavier, France). The membranes were incubated with anti-rabbit or anti-mouse Ab coupled to horseradish peroxidase (Sigma-Aldrich) then reacted with a chemiluminescent substrate (SuperSignal West Dura Extended Duration Substrate, Thermo Scientific), and revealed with G:BOX Chemi chemiluminescence imaging system (Bangalore, India).

### Histology and immunostaining

Groups of infected BALB and C.MBT-*Rvfs2* mice were euthanized at different times along the 14-day period of observation to monitor the development of RVF disease. A first group was euthanized at an early stage of infection, day 3 p.i. A second group was euthanized at the first clinical signs of illness which occurred on day 3 or 4 p.i. in C.MBT-*Rvfs2* mice, and between days 6 and 9 p.i. in BALB mice. Finally, BALB mice that survived until day 14 p.i. were also euthanized. Non infected BALB and C.MBT-*Rvfs2* mice were used as controls. The liver and brain were removed and immediately fixed for one week in 10% neutral-buffered formalin for biosafety reasons. Samples from each organ were embedded in paraffin; 4 μm-thick sections were cut and stained with hematoxylin and eosin (HE). Microscope slides were coded for blinded studies, and examined by a qualified veterinary pathologist (GJ). Non-quantitative immunohistochemical detection of the RVFV-infected cells was done using mouse antibodies against the N protein (dilution 1:100)^35^. A rabbit monoclonal antibody (Ref: AB16667, dilution 1:50; Abcam, Paris, France) was used to detect Ki67 antigen. Visualization was performed with the Histofine Simple Stain MAX-PO kit (Nichorei Biosciences Inc., Tokyo, Japan), a labeled polymer prepared by combining amino acid polymers with peroxidase and secondary antibody which is reduced to Fab’ fragment. This visualization procedure allows amplification of the positive signal and limitation of the background staining, especially when using mouse antibodies as for the detection of the RVFV. However, it does not allow quantitative evaluation of positive signals and intensity comparisons between samples.

### Primary hepatocyte preparation and infection

Seven to 12 week-old BALB and C.MBT-*Rvfs2* male mice were euthanized by cervical dislocation. Suspensions of hepatocytes were prepared as described in Li *et al*.^36^ using Collagenase type IV (PAN Biotech, Worthington, UK) at 100U/ml. Cells were cultured also according to Li *et al*.^36^. On the day after preparation, hepatocytes were infected with RVFV at an MOI of 3 for 1 hr. At 15, 24, 48 and 60 hours post-infection, the supernatant was collected for virus titration by plaque assay as above. Each condition was done in triplicate (3 wells).

### Bone marrow transplantation

Chimeric mice were produced by transplantation of bone-marrow cells after total body irradiation. We generated BALB mice reconstituted with C.MBT-*Rvfs2* marrow (C.MBT-*Rvfs2* → BALB chimeras) and C.MBT-*Rvfs2* mice reconstituted with BALB marrow (BALB → C.MBT-*Rvfs2* chimeras). Controls consisted of irradiated mice reconstituted with isogenic marrow, (BALB → BALB and C.MBT-*Rvfs2* → C.MBT-*Rvfs2* chimeras). Bone marrow cells (BMCs) were collected from both tibias and femurs of 5-6 week-old BALB or C.MBT-*Rvfs2* donor male mice. BMCs were resuspended in Hanks’ Balanced Salt Solution. After irradiation with one sub-lethal dose of gamma radiation (700 rad), 5-6 week-old BALB or C.MBT-*Rvfs2* recipient male mice received ~ 3 × 10^6^ BMCs in 0.15 ml by intravenous injection in the retro-orbital sinus. The extent of reconstitution was evaluated using a semi-quantitative PCR assay based on primers to *Apoptosis-associated tyrosine kinase* (*Aatk*) gene (Forward, 5′-CTACCCCAGGAGGACTGTGTCAGG-3′ and reverse 5′- GTCCTCCCCAACAATATCCTGGTGC-3′) that maps within *Rvfs2* interval. BALB and MBT alleles produce a fragment of 180 bp and 127 bp, respectively. Six weeks after the transplantation, the reconstitution in total peripheral blood of (BALB → C.MBT-*Rvfs2*) and (C.MBT-*Rvfs2* → BALB) mice was higher than 90%. At that time, bone marrow chimeras were infected intraperitoneally with 100 PFU of RVFV strain ZH548.

### Statistical analysis

Statistical analysis was performed using GraphPad Prism 6.0 (GraphPad Software, La Jolla, CA, USA) and R softwares. Mantel-Cox’s Logrank test was applied to assess survival curve differences. Two-way ANOVA was used to assess body weight and body temperature differences, with the two factors being the strains and the days post-infection. The p-values shown indicate the significance of the difference between strains. Mann Whitney-U test was used to analyze viral titers in serum and liver.

## Supplementary information

Supplementary Table S1.

## Data Availability

All relevant data are within the paper.
