# Supplementary material for: Genetic dissection of Rift Valley fever pathogenesis: Rvfs2 locus on mouse chromosome 11 enables survival to early-onset hepatitis
Source: Sci Rep. 2020 May 26;10:8734. doi: 10.1038/s41598-020-65683-w (PMC7250886; doi:10.1038/s41598-020-65683-w)
Supplement: Supplementary file 1 — Supplementary Table S1. [file 41598_2020_65683_MOESM1_ESM.pdf]

SUPPLEMENTARY INFORMATION

Manuscript title:

Genetic dissection of Rift Valley fever pathogenesis: *Rvfs2* locus on mouse chromosome 11 enables survival to early-onset hepatitis

Authors :

Leandro Batista,[1,2] Gregory Jouvion,[4,5] Dominique Simon-Chazottes,[1,3] Denis Houzelstein,[1] Odile Burlen-Defranoux,[6] Magali Boissière,[7] Satoko Tokuda,[1] Tania Zaverucha Do Valle,[1,8] Ana Cumano,[6] Marie Flamand,[7] Xavier Montagutelli,[1,3¶*] & Jean-Jacques Panthier[1¶]

	Infectious dose (PFU), IP					
	10		100		1000	
Inbred strain	Survival	Days of death	Survival	Days of death	Survival	Days of death
MBT/Pas	0/5	dpi 3-4	0/5	dpi 3-4	0/5	dpi 4
BALB/cByJ	4/5	dpi 13	0/5	dpi 7-10	0/5	dpi 7-10

Supplementary Table S1. Survival rate and days of death of MBT/Pas and BALB/cByJ 9-12 week-old male mice infected with 10, 100 or 1000 PFU of ZH548 RVFV (dpi : days post-infection).
